# Valorization of Agricultural Wastes into Environmentally Sustainable Asphalt Binders

**DOI:** 10.3390/molecules30173473

**Published:** 2025-08-23

**Authors:** Paolino Caputo, Valentina Gargiulo, Pietro Calandra, Valeria Loise, Luciana Cimino, Claudio Clemente, Aliya Kenzhegaliyeva, Yerdos Ongarbayev, Cesare Oliviero Rossi, Mikołaj Pochilskj, Jacek Gapinski, Michela Alfè

**Affiliations:** 1 Department of Chemistry and Chemical Technologies, University of Calabria, 87036 Rende, Italy; paolino.caputo@unical.it (P.C.); valeria.loise@unical.it (V.L.); cesare.oliviero@unical.it (C.O.R.); 2 CNR-STEMS, National Research Council, Institute of Sciences and Technologies for Sustainable Energy and Mobility, 80125 Napoli, Italy; valentina.gargiulo@stems.cnr.it (V.G.); luciana.cimino@stems.cnr.it (L.C.); claudio.clemente@unina.it (C.C.); michela.alfe@stems.cnr.it (M.A.); 3 CNR-ISMN, National Research Council, Institute for the Study of Nanostructured Materials, 00010 Montelibretti, Italy; 4 Department of Physics, University of Naples “Federico II”, 80126 Napoli, Italy; 5 Laboratory of Petrochemical Processes, Institute of Combustion Problems, Almaty 050012, Kazakhstan; aliakenzhik@gmail.com (A.K.); erdos.ongarbaev@kaznu.edu.kz (Y.O.); 6 Faculty of Chemistry and Chemical Technology, Al-Farabi Kazakh National University, Almaty 050040, Kazakhstan; 7 Faculty of Physics, Adam Mickiewicz University, 62-614 Poznan, Poland; mikolaj.pochylski@amu.edu.pl (M.P.); jacek.gapinski@amu.edu.pl (J.G.)

**Keywords:** waste biomass, biochar, valorization, bitumen modifier

## Abstract

The use of solid products deriving from the pyrolysis of wastes as potential substitute of traditional binders in asphalt preparation is investigated with the final goal of reducing production costs, preserving non-renewable resources, and promoting an effective resource use as well as recovery and recycling procedures, thus implementing a regenerative circular economy approach. Char derived from the pyrolysis of agricultural and aquaculture wastes has been explored as a novel alternative additive for asphalt production. Different feedstocks were used for the preparation of biochar by pyrolysis. The produced char samples, after an in-depth chemical and structural characterization, have been implemented in the preparation of asphalt mixtures, with their potential use as a binder evaluated by performing conventional rheological tests. To evaluate the potential anti-aging effect of char as an additive, bituminous formulations containing 3 to 6 wt.% char were subjected to short-term simulated aging using the Rolling Thin-Film Oven Test (RTFOT) method. The resulting mechanical properties were then assessed. The results indicate that the all the tested char samples have limited modifying properties towards the gel-to-sol transition temperature. Among the samples, lemon peel-derived char (LP-char) showed superior antioxidant properties against bitumen oxidative aging. This study suggests that certain chemical characteristics can serve as predictive indicators of antioxidant activity in biochars produced from biomass pyrolysis.

## 1. Introduction

In road-paving preparation processes, great effort is focused on improving bitumen’s properties, essentially its mechanical properties, through the use of specific additives. To meet the recent needs dictated by the search for a sustainable future, materials originating from renewable sources have become prevalent: bio-modifiers [1,2], bio-fillers in asphalt mixtures [3], bio-rejuvenators [4,5], and bio-adhesion promoters [6] are just examples representing an important recent trend. In this framework, four main categories can be identified: (i) bio-oils derived from biomass pyrolysis that can improve the viscosity and low-temperature properties of bitumen [7]; (ii) bio-asphaltenes extracted from bio-oils or bio-based sources that can be used as modifiers for bitumen to improve its performance [8]; (iii) bio-surfactants derived from renewable sources, such as plant-based oils, that can be used to modify the surface properties of bitumen and improve its adhesion to aggregates [9]; and (iv) bio-polymers like cellulose derivatives, starches, or lignin that can be added to bitumen to improve its elasticity, moisture resistance, and aging properties [10]. This scenario exhibits a substantial complexity, and the high cost of fossil fuel is pushing the field towards a highly relevant and increasingly discussed topic.

Among the emerging materials, biochar is one of the most promising bitumen modifiers [11], as testified by the very recent research articles [12,13,14,15,16,17,18] and literature reviews [19,20]. Biochar is the solid product of the thermal degradation (pyrolysis) of biomass [21]. From a chemical point of view, the main characteristics of biochar are the high carbon content, the high value of the specific surface area, high porosity, low thermal conductivity values, particles of small dimensions, extensive variation in the functional moieties decorating the particle surface, long-term stability, and low flammability [22,23]. In comparison to activated carbon, biochar offers a more cost-effective and environmentally friendly alternative, as it is produced under milder thermal conditions (up to 700 °C instead of 800–1000 °C typical of activated carbon production) and heating rates (below 100 °C/min) than activated carbons [11] and in the absence of physical (ex. steam, CO_2_) or chemical activating agents (ex. KOH, ZnCl_2_, H_3_PO_4_).

The rheological properties of bitumens, namely viscosity, failure temperature, aging resistance, stiffness, and rutting resistance, can be affected by biochar addition, even when the added amounts are small (below <10 wt.%) [13,14,16,17]. This is due to the high surface-to-volume ratio of its particles and the tunable surface properties [11,15]. However, it must be noted that, as shown by the pioneering study by Zhao et al., the effect of biochar addition (amounts ranging between 5 and 10 wt.%) in promoting a bitumen viscosity increase is larger at high service temperatures rather than at low service ones (≤10 °C) [24].

In the field of novel bitumen additives, alternative binders/additives acting as antiaging agents can mitigate negative effects created by conventional bitumen and can ensure longer-lasting road pavement, thus reducing maintenance costs and lengthening the time until a replacement is needed. This addresses the future requirements of bitumen in terms of sustainability and circular economy for pavement construction [25].

This work explores the potential use of biochar obtained through the pyrolysis of agricultural and aquacultural wastes as a modifier in bitumen preparation for road applications. Biomasses from various sources have been used to produce different biochars by pyrolysis, which were then individually incorporated into bitumen to compare the resulting properties. For better comparison, the chars have different specific characteristics: a high aromatic char is obtained by the pyrolysis of alkali lignin; N-rich chars were obtained by chitosan and thistle (*Cirsium vulgare*), while O-rich chars were obtained by lemon peels and pectin from citrus fruits.

This study evaluates whether the addition of the selected chars to a bitumen can improve mechanical performance and resistance to aging, with the intention of exploring the integration of urban waste into asphalt cycles by optimizing the use of pyrolysis products in bitumen preparation [26]. Our primary aim is to investigate innovative reuse strategies and their conceptual relevance, establishing the basis for subsequent environmental and economic assessments in future research phases.

## 2. Results

### 2.1. Rheological Properties

**Figure 1:**
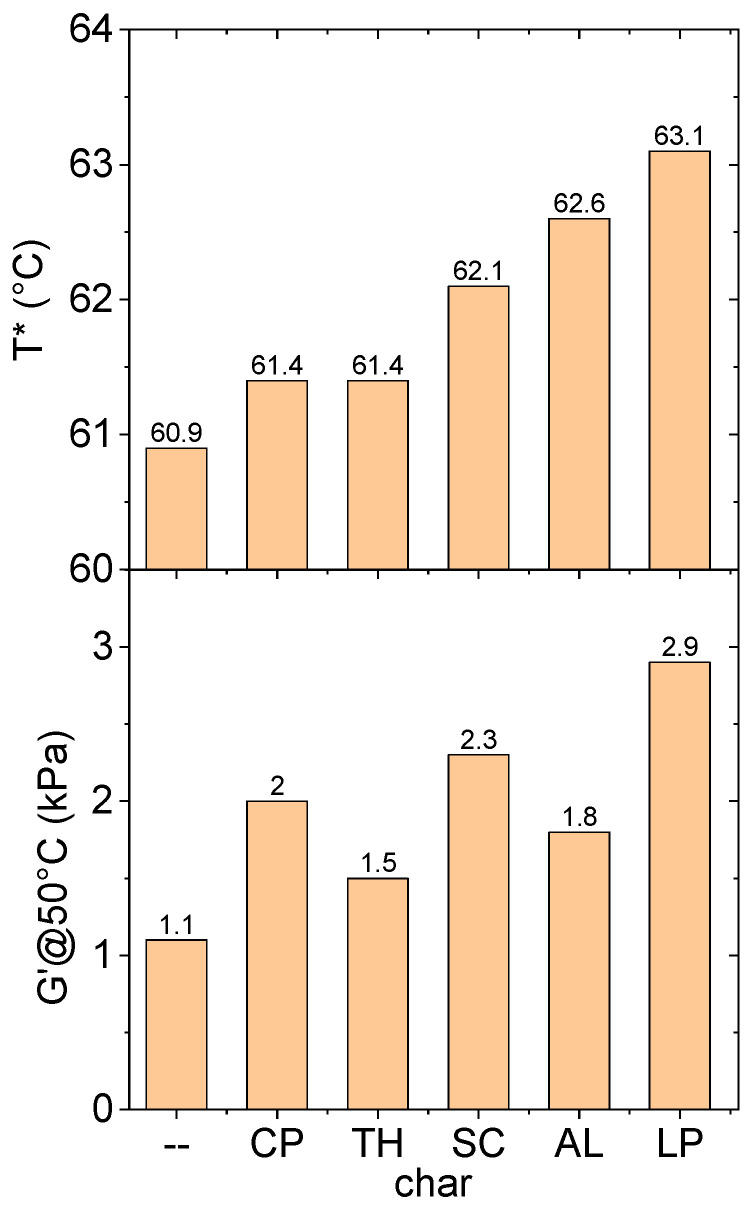
T* and G′@50°C values for the various char-modified bitumens. The first column corresponds to the neat bitumen.

In rheology experiments, the storage modulus (G′) and the loss modulus (G″) are recorded as a function of temperature (frequency was kept fixed at 1 Hz). G′ represents the in-phase component of the mechanical (complex) modulus, i.e., the elastic energy gained by the system under oscillatory deformation; on the other hand, G″ is the out-of-phase component, reflecting the irreversible dissipation of mechanical energy under the same deformation. Under increasing temperature, the samples monotonously soften, with a parallel continuous decrease in G′. At a certain, material specific temperature, the material cannot elastically sustain any deformation and G′ tends to zero, so the material practically becomes a Newtonian fluid. This is a real gel-to-sol transition, and the corresponding temperature (T*) has been used as an indicator of the resistance to temperature changes in our samples. The values are reported in Figure 1. Another key parameter examined in our study is the G′ of the sample measured at 50 °C (G′@50°C), i.e., a temperature usually regarded as representative of typical service conditions [27]. It can be therefore considered as the rigidity under typical working conditions of the bituminous material. G′@50°C values are also reported in Figure 1.

Two noteworthy points emerge from the analysis:

All chars cause an increase in T*. This is somewhat expected, since the insertion of carbonaceous particles within the bituminous matrix, which has the same organic carbon-based nature, causes the establishment of char–bitumen interactions and therefore a stronger network made by bitumen–char–bitumen is formed. The interconnected network exhibits enhanced thermal resilience, thereby leading to an increase in T*. The most evident effect is shown by the LP-char which is able to induce an increase of 2.2 °C in T*. A comparison with CaCO_3-_added bitumens can be useful (CaCO_3_ is a conventional inert filler used as a benchmark in most published research works on bitumen and asphalt concretes). In fact, even the addition of inert CaCO_3_ to bitumen results in a robustness increase and thus in an increase in T* [28]. It is worth noting, however, that the addition of 3 wt.% CaCO_3_ causes a 4 °C increase in T*.

The effect of our chars instead is lower. This suggests that the integration of the char does not significantly disrupt the molecular arrangement of the bituminous material. This can be considered a significant finding from an environmental standpoint, since bitumen (and asphalt concrete) is potentially able to incorporate huge amounts of chars without modifying its rheological properties much.

Similar conclusions can be drawn when evaluating the parameter G′@50°C. All chars cause an increase in G′ at this temperature. Such reinforcement, reflected by a higher storage modulus G′, is generally expected when solid particles are incorporated into bitumen. However, the magnitude of this effect is less pronounced than that observed with the addition of CaCO_3_ to a bitumen, which generally causes a 3-fold increase in G′@50°C, a variation reached only by LP-char, which is confirmed to be the strongest modifier among all the chars studied in this work. Here, from a different perspective, it is confirmed that the addition of the proposed chars does not modify the bitumen’s rheological properties much.

**Figure 2:**
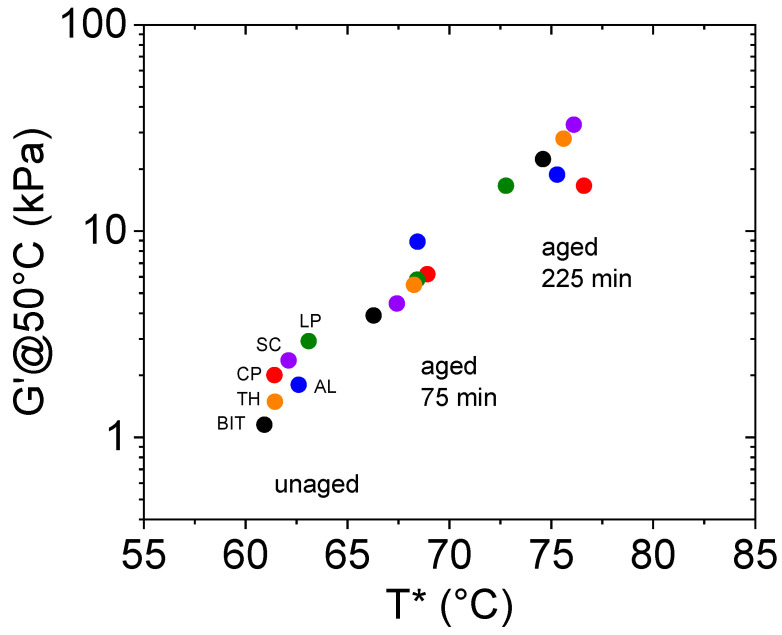
The correlation between G′@50°C and T* values for char-modified bitumens. The char contained in the bitumen is indicated with a label, and coloring is consistent for unaged and aged. Neat bitumen (BIT) was also reported for comparison.

Interestingly, we found that G′@50°C correlates well with T* (see Figure 2). Our data are in accordance with the literature data from other bituminous samples modified by chars coming from pyrolysis [29].

Since G′@50°C and T* are two independent parameters, their correlation suggests that the conclusions derived by their analysis not only mutually reinforce but probably could be considered as different aspects of the same inner phenomenon.

**Figure 3:**
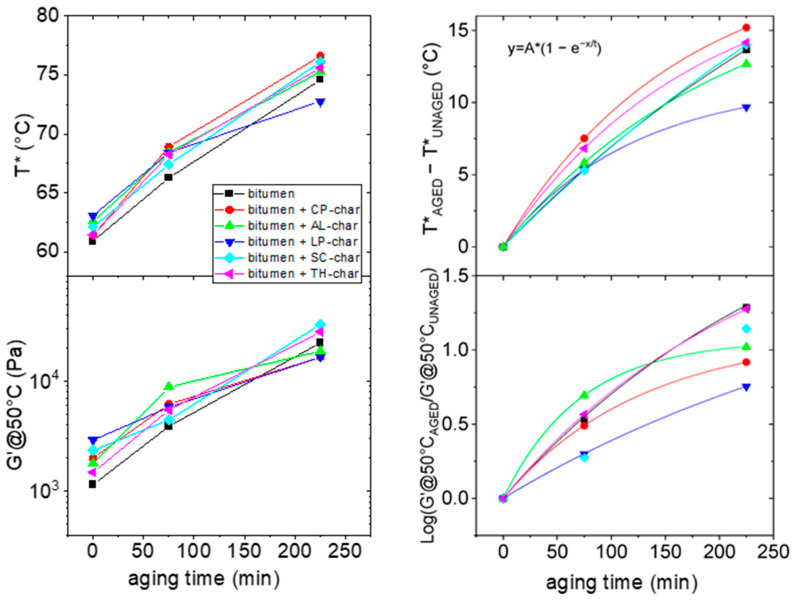
T* and G′@50°C values as a function of aging time (**left panel**). Derived variations in parameters (**right panel**).

Another important observation arises from the comparison between the modified unaged samples and those subjected to simulated aging for 75 and 225 min. An agent is referred to as antioxidant if it reduces the effect of oxidative aging on the bitumen [30]. In practice, this happens when it prevents the increase in the T* (or G′@50°C) upon aging. To better show the influences of the various chars upon aging, T* and G′@50°C are reported as a function of aging time in the left panel in Figure 3.

The data presented in this figure indicates that the bitumen modified with LP-char exhibits a measurable antioxidant effect. Interestingly, this is shown consistently by both T* and G′@50°C.

However, for clarity, we observed the following, shown in the right panel:

(1) The increase in transition temperature (ΔT = T*_AGED_ − T*_UNAGED_) after a given aging tune, i.e., the values obtained from differences between the T* recorded after and before the aging process RTFOT under short (75 min) and long (225 min) times.
(2) The increase in the G′@50°C value, calculated as the logarithm of the quantity (G′@50°C_AGED_/G′@50°C_UNAGED_) i.e., the ratio of G′@50°C recorded after and before the aging process RTFOT under short (75 min) and long (225 min) times.

The data are connected by the exponential growth curve, whose function is reported in the same figure, connecting the three points for each sample. From this representation it is now clear that the bitumen modified with LP-char has an antioxidant effect, possessing the least (in magnitude) and the slowest variation in T* and in G′@50°C, respectively.

Given the correlation between T* and G′@50°C, the joint conclusion derived from these parameters aligns well with the broader findings of the analysis.

To understand the reason, at the microscopic level, for the peculiar behavior of LP-char, further analyses were carried out on the chars to highlight their physicochemical characteristics.

### 2.2. Char Characterization (Morphology)

All char samples exhibit an aggregated structure, comprising millimeter and sub-millimeter aggregates. These aggregates are not monolithic but consist of smaller primary particles held together by weak physical interactions, as evidenced by their facile disaggregation during handling. This behavior is characteristic of carbonaceous materials derived from pyrolysis, where van der Waals forces and surface interactions dominate particle cohesion [31]. During bitumen–char composite fabrication, the combination of thermal energy (150 °C) and mechanical shear during bitumen mixing promotes the disintegration of these aggregates, yielding a homogeneous char–bitumen composite devoid of macroscopic particles, as witnessed by the homogeneous texture of the final char-containing bitumen.

To quantify the primary particle-size distribution, the chars were dispersed in silicone oil—a surrogate medium chosen to mimic the apolar environment of bitumen [32]. The high viscosity of silicone oil at room temperature (~350 mPa·s) approximates the rheological conditions of bitumen at mixing temperatures [33], while its chemical inertness prevents artificial particle modification. Mild sonication was employed to accelerate dispersion, simulating the shear forces encountered during bitumen processing. This approach aligns with recent methodologies for characterizing nanofiller dispersion in polymer matrices, where sonication parameters are controlled to avoid over-fragmentation [34]. In any case, our study is comparative, ensuring that all the samples have undergone the same treatment, with strict control and reproduction of all the operational aspects. Optical microscopy of the resulting suspensions revealed the intrinsic particle-size distribution, though it is important to note that this ex situ analysis provides only a proxy for the true state of dispersion within the bitumen matrix: direct observation of char particles in the bitumen phase would require advanced techniques such as cryo-electron microscopy or synchrotron X-ray tomography, which preserve the native microstructure [35,36]. Nevertheless, the silicone oil dispersion method offers a pragmatic first approximation for assessing particle deagglomeration tendencies under processing conditions, as already exploited in previous works [29].

**Figure 4:**
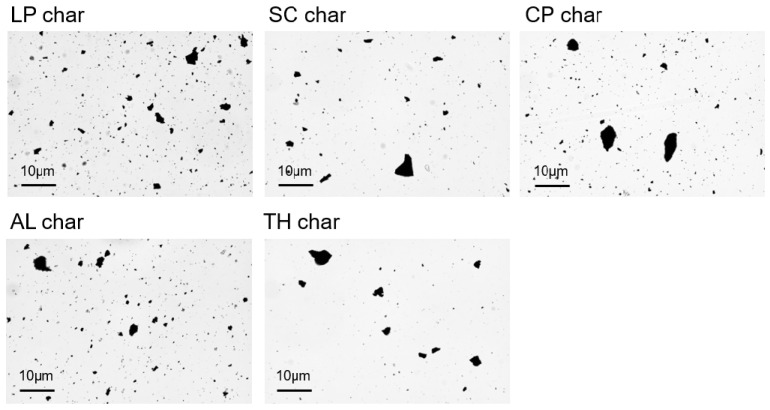
Representative microphotographs for the various chars.

Representative microphotographs of the char are shown in Figure 4. It can be seen that polydispersed and isolated particles are present. They are generally globular but with slightly irregular shapes and are randomly distributed all over the sample.

**Figure 5:**
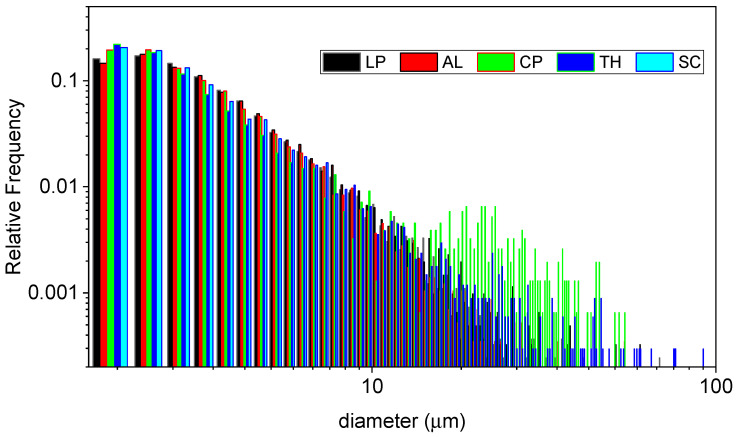
Size distributions for the various chars.

The particle-size distribution function is reported in Figure 5.

In our analysis of the size distributions, it is important to observe that the minimum resolvable distance, i.e., resolution (r), is

$$r=\frac{0.6\lambda }{NA}$$

where NA is the numerical aperture of the objective we used (NA = 0.25) [37], giving a value of 1.2 µm. This means that our experiments can probe particles bigger than this value, being somehow blind to sub-micrometer sized particles.

As can be seen, all the samples resemble similar size distribution, with the following peculiarities:

- The CP-char sample shows the presence of a bump in the range 20–50 µm suggesting the tendency towards a bimodal distribution. Even if the relative abundance of the bigger particles is limited, being less than one order of magnitude lower than the smaller ones in number, it must be noted that in terms of mass, such bigger particles constitute the majority of the sample. The volume indeed scales with the third power of the diameter, so the bigger particles are ~10 times larger and consequently have a volume ~1000 times higher than the smaller ones, a difference that outweighs their lower abundance.
- The TH-char sample shows a slightly broader size distribution. The presence of bigger particles whose diameter can extend up to 100 µm is notable, as well as the consequently slightly lower frequency of occurrence of particles in the range 3–10 µm in the size distribution. Quantitatively, the polydispersity, measured as the standard deviation of the diameter values, is around 8 when compared to the values for the other samples ranging in the 4–6.5 interval.

It is important to note that the samples showing this peculiarity are among the most stable ones, as seen by thermogravimetry (see the next section). These aspects could be seen as mutually correlated in a rational vision of our samples; after all, it is reasonable to suppose that the more stable the material the lower the degree of fragmentation during the pyrolysis.

**Figure 6:**
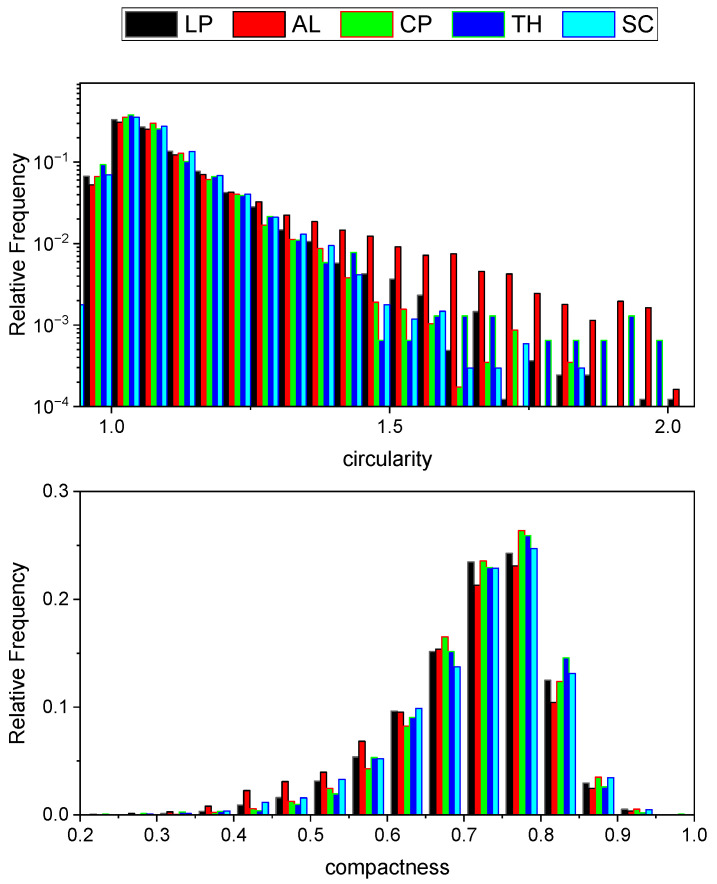
Circularity and compactness distributions for the various chars.

Another noteworthy observation emerges from the analysis of the circularity factor and the compactness distributions. It is important to recall that circularity, also referred to as the Heywood circularity factor, is defined as the ratio between the experimentally measured perimeter of a particle and the circumference of a circle having an equivalent area. The closer the shape of a particle is to a disk, the closer the Heywood circularity factor is to 1. As for the compactness factor, it is another independent parameter and is derived as the area of the particle divided by the area of the Bounding Rectangle. The compactness factor clearly belongs to the interval [0, 1]. The two graphs are reported in Figure 6.

As can be seen by inspecting Figure 6, the graphs show higher circularity and lower compactness for AL-char. As illustrated by the Heywood circularity parameter distribution function, which for this sample shows less frequently low values, but more frequently high values, with respect to the other samples. This is the reflection of the fact that the particles are more irregular in shape and generally more elongated. Coherently, for the same reason, particle compactness generally decreases. This is reasonably due to the high content in lignin, an inherently anisotropic molecule which confers its anisotropy to the final material. It must also be noticed that in some cases, connected particles with a chain-like feature (see Figure 4) can also be seen. It is not clear, with the present data, if it is the consequence of a higher probability for more elongated particles dispersed in the suspension to come into contact, and therefore interact with each other, or instead whether chemical reasons should be considered.

However, the most interesting result of the microscopy is that the peculiar effect of LP-char in modifying the bitumen properties seems to not be a result of morphologic effects. Although some differences in the size and shape distributions are present among the various chars, the particle morphology is not a consequence of the antioxidant effect of LP-char, so the effects of chemistry need to be considered. They are explored in the next section.

### 2.3. Char Characterization (Thermal Behavior, Chemical Composition, and Contact Angle)

**Figure 7:**
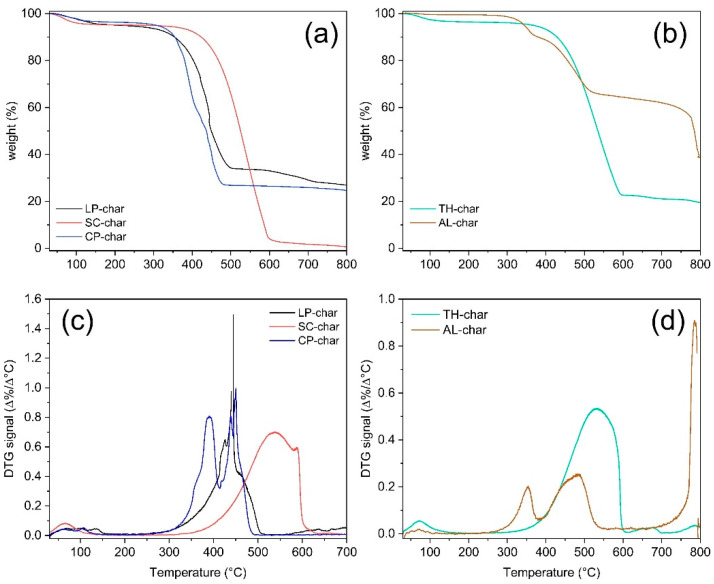
TG and DTG curves of char samples (air, 40 mL/min, HR = 10 °C/min). Panels (**a**,**c**) report TG profiles and DTG curves of LP-char, CP-char, and SC-char. Panels (**b**,**d**) contain TG profiles and DTG curves of AL-char and TH-char.

C, H, and N contents of all the char samples are listed in Table 1. Figure 7 reports the TG behavior of the biochar samples under an oxidizing atmosphere. The information deriving from these analyses are being discussed jointly.

LP-char is characterized by a quite high C content (55.9 wt.%) and low H and N contents (0.65 wt.% and 0.35 wt.%, respectively), and since it completely decomposes before reaching 500 °C under an oxidizing atmosphere, it is considered one of the less thermally stable chars under study. Such a tendency to decompose at low temperature can be explained by taking into account the possible catalytic effects promoted by the inorganic species present in the starting feedstock and thus in the corresponding char (see the high ash content in Table S1 and the high value of other elements reported in Table 1). SC-char contains the highest C and N contents, 70.8 wt.% and 8.5 wt.%, respectively; this characteristic reflects the composition of the parent feedstock (see Table S2 of Supplementary Materials). SC-char is also characterized by an overall structural uniformity and a quite good degree of graphitization, as indicated by its TG profile exhibiting only one main thermal event around 550 °C; homogeneously structured carbon-based materials such as activated carbons and combustion-derived materials are usually characterized by burn-off temperatures above 550 °C [38]. CP-char is composed mainly of carbon (62.8 wt.%), since its hydrogen content is very low (0.2 wt.%) and that of nitrogen can be considered negligible. The content of other elements is also consistent (37 wt.%), as expected based on the composition of the starting feedstock (see Tables S1 and S2 of Supplementary Materials). Despite the high carbon content, as it decomposes at a temperature below 350 °C, CP-char is the least thermally stable char investigated. This low thermal stability is probably a consequence of catalytic effects exerted by inorganic species already present in the starting feedstock (4.53 wt.% of ashes in the parent feedstock, Table S1). TH-char contains a C content of 56.4 wt.%, and quite high H and N contents (0.80 wt.% and 1.60 wt.%, respectively). Its burn-off temperature is around 550 °C, comparable to that of some activated carbons and combustion-derived materials [38], which leads us to classify TH-char as a material with a good degree of graphitization. AL-char is characterized by a quite high C content (57.7 wt.%), a quite low H content (0.70 wt.%), and by the absence of N. The TG profile of AL-char is the more complex due to the presence of three thermal events: one is ~300 °C with a low intensity ascribable to the decomposition of side groups, one is ~450 °C ascribable to the carbonaceous network burn-off, and one above 750 °C is ascribable to the decomposition of inorganic species. The decomposition temperature of the carbonaceous network is below 450 °C, probably because the inorganic species present in the char sample promote catalytic phenomena (for details on inorganic species, see Table 1, Tables S1 and S2 of the Supplementary Materials, where the ash content of the parent feedstock is reported).

The presence of inorganic compounds has mainly been found in AL-char, LP-char, TH-char, and CP-char. Through infrared spectroscopy (FT-IR), low-intensity bands below 1000 cm^−1^, ascribable to vibrational modes of inorganic components, were found. Through X-ray diffraction (XRD), sharp peaks at 2θ > 25°, ascribable to calcite (CaCO_3_), were found in LP-char and in TH-char, while natrite (Na_2_CO_3_) was found in AL-char and K_2_CO_3_ in CP-char. For a detailed analysis of FT-IR and XRD data, refer to the supporting information (Figures S2 and S3).

The peculiar behavior of LP-char can be then traced back to its chemical nature and structure: the low H content and the high C content and other elements (Table 1), as compared to the other char samples, make this char a better fit for incorporation in the bituminous structures exerting an antioxidant effect. It can also be argued that the significant presence of other elements (different from C, H, and N) can also further help in establishing intermolecular interactions with asphalthenes, and their aggregates at the various levels of complexity [39], via their heteroatoms.

To better understand the char–bitumen interactions, a determination of the hydrophilicity and lipophilicity of char samples has been carried out by the measurement of contact angle (CA) of each char sample. Hydrophilicity and lipophilicity are indeed crucial surface properties in material, influencing the interaction of materials with water and oil, respectively: hydrophilic materials exhibit a strong affinity for water molecules, facilitating water absorption and spreading; in contrast, oleophilic materials demonstrate a preference for oil molecules, promoting oil absorption and retention. Understanding and quantifying these surface properties is essential for interpreting the interactions between char and the hydrophobic and hydrophilic components of bitumens.

The CA values reported in Table 2 indicate that all the investigated char samples exhibit an oleophilic behavior, with a low oil CA values. LP-, AL-, and CP-derived char samples also exhibit a hydrophilic character with low water CA values, indicative of an amphiphilic surface characteristic.

SC-char and TH-char are clearly oleophilic and hydrophobic materials. Of note, the oil absorption rate also depends on the microstructure and surface chemistry of each material. In the case of SC-char, the oil is not immediately absorbed on its surface, and resists for a longer period before being completely absorbed. This behavior could be due to various factors, such as surface roughness or the morphology of the materials, which might prevent rapid absorption or favor a less immediate surface interaction. As confirmation of this, the roughness measurements (Ra) for SC-char and TH-char are 3.3 and 1.95, respectively, values which are higher compared to those of the other samples.

In conclusion, the findings suggest that in LP-char, owing not only to its chemical composition but also to other contributing factors, is well-suited for incorporation into bituminous matrices and for establishing interactions with asphalthenes, and the interactions with asphalthenes can be facilitated by the low LP-char hydrophobicity.

## 3. Materials and Methods

### 3.1. Materials

Alkali lignin (AL) (CAS number: 8068-05-1), citrus pectin (CP) (CAS number: 9000-69-5), and shrimp-derived chitosan (SC) (CAS number: 9012-76-4) were purchased form Merck KGaA (Darmstadt, Germany) and used as received. Lemon peels (LP) were kindly provided by Piemme s.r.l (Piano di Sorrento (NA), Italy) after their use in an ethanol extraction process to produce Limoncello liquor. Prior to use, the lemon peels were dried in an oven at 80 °C for 24 h to remove traces of alcohol and water and then they were milled in a blade chopper up to a micrometric size. Dried thistle (TH) was kindly provided by Agriculture Department of University of Naples “Federico II” and used as received. Details about feedstock composition and thermal behavior are reported in the Supplementary Materials section as Tables S1 and S2 and as Figure S1, respectively.

A low-penetration-grade bitumen (50/70) produced in Saudi Arabia and kindly provided by Lo Prete (Terranova Sappo Minulio (RC), Italy) was used in this study as neat bitumen (BIT).

### 3.2. Biochar Production

AL, LP, TH, SC, CP were carbonized in a LECO 701 thermobalance at 550 °C under an inert atmosphere (nitrogen, flux 8.5 L/min). The thermal treatment was carried out as follows: the temperature was first raised from 25 °C up to 100 °C (heating rate (HR) of 15 °C/min) with a holding time of 10 min to remove moisture; after that, it was raised up to 550 °C (HR of 37 °C/min) and then held for 1.5 h. In the end, the system was cooled down, maintaining an inert atmosphere (nitrogen flux 5.0 L/min).

### 3.3. Preparation of Bitumen–Char Composites

Firstly, bitumen was heated in oven at 160 °C until fully melted. Then, 100 g of bitumen was transferred onto a hot plate maintained at 150 °C and biochar was added in amounts ranging from 3 to 6 wt.%. The samples were mechanically stirred at 700 rpm for 1 h. The resulting samples were poured into small, sealed containers and then stored in a dark, temperature-controlled chamber at 25 °C to preserve their morphology.

### 3.4. Aging Tests

To replicate the natural aging process of bitumen, the Rolling Thin-Film Oven Test (RTFOT) was conducted in accordance with ASTM D2872-04, which is in agreement with the guidelines provided in UNI EN 12607-1. This procedure simulates bitumen hardening caused by heat and air exposure, utilizing a double-walled oven equipped with an internal fan to circulate air at a controlled temperature of 163 °C. During the test, a thin bitumen layer (around 1.25 mm thick) is exposed to a stream of hot air for 75 min. In addition to the standard protocol, extended exposure experiments (225 min) were also performed to mimic more advanced aging conditions [40,41].

### 3.5. Structural Characterization Methods

Rheological measurements were carried out by a dynamic stress-controlled rheometer (SR5000, Rheometric Scientific, Piscataway, NJ, USA) in a parallel-plate configuration. The gap between the plates was 2 mm and the diameter was 25 mm. The frequency was kept fixed at 1 Hz and the temperature was varied by a Peltier element with an accuracy of ±0.1 °C. The heating rate was 1 °C/min. The setup and related conditions matched previous studies [42]. To ensure a linear response, preliminary stress-sweep tests were performed [43], i.e., taking measurements at the stress value at which the material response is linear. Throughout the taking of measurements, small-amplitude oscillatory shear conditions were maintained. All rheological measurements were performed in triplicate, with a standard deviation (SD) below 2%.

Optical microscopy was conducted by dispersing the chars in silicone oil at a concentration of 0.1 wt.%. These char-in-oil chemical systems were designed as models for bituminous matrices containing particles of char derived from pyrolysis. Transmission optical microscopy under these conditions enabled the characterization of the char as though it was embedded as a filler within the bituminous matrix. Gentle bath sonication through a low-power commercial sonicator prior to sample preparation (a duration of about 40 min) was carried out for each sample. Four droplets of 10 μL each were deposited closely onto a quartz plate, making part of a UV cell of 0.1 mm path length, and then the other plate was placed on top to close the cell. The liquid spanned the cell, so the predetermined thickness of 0.1 was guaranteed. The cell was horizontally placed on a microscope Olympus BX53 (EVIDENT Corporation, Tokyo, Japan) equipped with a Nikon 20× magnification and 0.25 numerical aperture objective. The camera used was an Olympus DP23 EVIDENT Corporation (Tokyo, Japan). Given the relationship between the numerical aperture (NA) and depth of focus (DOF) [44]

$$DOF=\frac{\lambda }{{2\left(NA\right)}^{2}}$$

where λ is the wavelength of light (for visible light, the mean value of ~500 nm can be assumed), it can be observed that the DOF is about 4 µm. For this reason, the sample was allowed to settle down overnight to allow all the particles to be deposited at the bottom of the cell. Preliminary observations showed that char particles in silicone oil typically settle within a few hours; however, to ensure thorough deposition, the sample was allowed to rest overnight, and microscopic observations were conducted the following day. About thirty pictures were captured from various regions of the sample. Focusing at the bottom of the cell and at different heights confirmed that practically all particles were deposited at the bottom. The total number of observed particles turned out to be always more than 5000, guaranteeing good statistics.

Images were analyzed using specialized LabVIEW-based software for particle recognition. The areas of the particles were then extracted, as well as their circularity, defined as the ratio of the perimeter of the particle divided by the circumference of the circle with the same area as that particle, or, in other words, the Heywood circularity factor used in some papers [45].

The Waddel Disk Diameter (i.e., the diameter of a disk with the same area as the particle, hereafter simply called “diameter”), the perimeter of the equivalent circle, the convex hull perimeter, the convex hull area, the compactness factor, and factor-type distributions were also evaluated.

Proximate analysis for feedstock humidity, volatile, ashes, and fixed carbon-contents determination was performed on a LECO 701 thermobalance according to ASTM D7582-15 (Standard Test Methods for Proximate Analysis of Coal and Coke by Macro Thermogravimetric Analysis). Each measure was repeated thrice and the results expressed as the mean value of the three independent measurements.

C, H, N contents were estimated by ultimate analysis in accordance with ASTM D3176-15 (Standard Practice for Ultimate Analysis of Coal and Coke), by using a LECO 628 analyzer after EDTA calibration and performing measurements in triplicates.

ICP-MS analysis was performed on an Agilent ICP-MS 7500ce spectrometer. Prior to ICP-MS analysis, digested solutions of each feedstock were prepared as follows: 100 mg of each feedstock were suspended in deionized water and digested with HNO_3_ (65%) and H_2_O_2_ (30%) by microwave heating in accordance with US-EPA 3051 and 3052 methods. Each digested sample was filtered, diluted with deionized water, and analyzed. Each measurement was repeated thrice.

The thermal behavior of the starting feedstocks and char samples was investigated by thermogravimetry on a Perkin–Elmer STA6000 thermobalance. The feedstocks were heated up from 30 °C up to 700 °C, applying a HR of 5 °C/min under an inert atmosphere (N_2_, 40 mL/min), while char samples were heated from 30 °C up to 700 °C applying a HR of 10 °C/min under an oxidizing atmosphere (synthetic air, 40 mL/min). For each measurement, an amount of material ranging between 5 and 20 mg was loaded into an alumina crucible that had previously been thermally treated in a furnace at 920 °C to ensure an accurate determination of the solid residue.

The surface chemistry of the char samples was investigated by infrared spectroscopy. FT-IR spectra in the 450–4000 cm^−1^ range were acquired on a Perkin–Elmer Frontier MIR spectrophotometer operated in transmittance mode using KBr pellets (2 wt.%), collecting 8 scans and correcting the background noise.

X-ray powder diffraction (XRD) analyses were carried out to evaluate the crystallinity of char samples. XRD patterns were acquired in the 2θ range 3–90° using a Rigaku Miniflex 600 automated diffractometer equipped with CuKα radiation source. Crystal-phase attribution was performed using PDF-5 2024 (International Centre for Diffraction Data, Tokyo, Japan) database.

Contact-angle (CA) measurements were performed on chars gently pressed onto a glass slide, using the DataPhysics OCA 50 Package 2 apparatus. Deionized water was used for hydrophobicity/hydrophilicity analysis, while a vegetable oil was employed for oleophilicity/oleophobicity analysis. The liquids were dispensed dropwise onto the chars, and the drop profiles were captured using a CCD camera. The contact angles were then evaluated based on the extracted images.

The surface roughness value Ra of the chars pressed onto a glass slide was measured using a DektakXT Stylus Profiler (Bruker, Billerica, MA, USA) equipped with a 2 μm tip stylus. Six single-line measurements, each 20 mm in length, were conducted to determine the Ra value.

## 4. Conclusions

In this work:

- Biochars derived from selected biomass sources through pyrolysis have been structurally characterized, resulting in the identification of key properties for each char: alkali lignin yielded high-aromatic char, shrimp chitosan and thistle (*Cirsium vulgare*) produced nitrogen-rich chars, and lemon peel and citrus pectin produced oxygen-rich chars;
- All the produced chars have been tested as bitumen modifiers and antioxidant agents;
- All the tested chars were found to have limited modifying properties towards the gel-to-sol transition temperature, resulting in an increase of only 0.5 to 2.5 °C, significantly lower than the ~4 °C increase observed with CaCO_3_ addition.
- Among the samples, lemon peel-derived char (LP-char) showed superior antioxidant properties against bitumen oxidative aging: after a long aging time (225 min), the T* of the bitumen loaded with LP-char is ~10 °C higher than that of the unaged sample, conversely, that of the other char-loaded bitumen samples is above 13 °C;
- The antioxidant effect of LP-char was found to not be primarily related to particle morphology (size and shape), but instead associated with chemical attributes, including lowest hydrogen content, high carbon and inorganic elemental contents, and lowest hydrophobicity, which enhanced the interactions with the bituminous molecular matrix;
- A key insight of this study is that specific chemical characteristics may serve as predictive indicators of antioxidant activity in biochars derived from biomass pyrolysis.

Although the current dataset remains limited and does not encompass a multi-dimensional evaluation of biochar’s performance as a binder modifier, it may be helpful as a starting point to identify and isolate specific and key characteristics that influence its functionality and guide future investigations, including an environmental and economic assessment, also taking limiting factors linked to biomass use into consideration (e.g., feedstock variability, process scalability, long-term field performance).

## Figures and Tables

**Table 1:** The results of the final analysis of char samples.

	C (wt.%)	H (wt.%)	N (wt.%)	Other Elements (wt.%) *
LP-char	55.9	0.65	0.35	43.1
AL-char	57.7	0.70	-	41.6
CP-char	62.8	0.2	-	37.0
SC-char	70.8	1.50	8.50	19.2
TH-char	56.4	0.80	1.60	41.2

* Evaluated by difference to 100.

**Table 2:** Contact angle (CA) and average roughness (Ra).

	Lipophilicity/ Lipophobicity (CA, °)	Hydrophilicity/ Hydrophobicity (CA, °)	Average Roughness (Ra, µm)
LP-char	51.96	28.90	0.44
AL-char	29.19	42.79	0.57
CP-char	45.35	35.04	0.23
SC-char	57.73	101.21	3.3
TH-char	25.89	132.83	1.95

## Data Availability

Dataset available on request from the authors.

## Supplementary Materials

The following supporting information can be downloaded at: https://www.mdpi.com/article/10.3390/molecules30173473/s1, Table S1: Feedstocks proximate analysis results; Table S2: Feedstocks composition; Figure S1: TG and DTG curves of the feedstocks (N_2_, 40 mL/min, HR = 5°C/min). Panel a and c TG profiles and DTG curves of polysaccharidic-rich feedstocks (LP, CP and SC). Panels b and d TG profiles and DTG curves of lignocellulosic based feedstocks (AL and TH); Figure S2: XRD patterns of char samples. Figure S3: FTIR spectra of char samples. The spectra are height-normalized and vertically shifted. Reference [46] is cited in supplementary materials.
