# Supplementary material for: Valorization of Agricultural Wastes into Environmentally Sustainable Asphalt Binders
Source: Molecules. 2025 Aug 23;30(17):3473. doi: 10.3390/molecules30173473 (PMC12430698; doi:10.3390/molecules30173473)
Supplement: Supplementary file 1 [file molecules-30-03473-s001.zip › molecules-3724803-supplementary.pdf]

SUPPORTING INFORMATION

# Valorization of Agricultural Wastes into Environmentally Sustainable Asphalt Binders

**Paolino Caputo [1], Valentina Gargiulo [2], Pietro Calandra [3,*], Valeria Loise [1], Luciana Cimino [2], Claudio Clemente [2,4], Aliya Kenzhegaliyeva [5,6], Yerdos Ongarbayev [5,6], Cesare Oliviero Rossi [1], Mikołaj Pochilskj [7], Jacek Gapinski [7] and Michela Alfè [2]**

1 Department of Chemistry and Chemical Technologies, University of Calabria, 87036 Rende, Italy; paolino.caputo@unical.it (P.C.); valeria.loise@unical.it (V.L.); cesare.oliviero@unical.it (C.O.R.)
2 CNR-STEMS, National Research Council, Institute of Sciences and Technologies for Sustainable Energy and Mobility, *80125* Napoli, Italy; valentina.gargiulo@stems.cnr.it (V.G.); luciana.cimino@stems.cnr.it (L.C.); claudio.clemente@unina.it (C.C.); michela.alfe@stems.cnr.it (M.A.)
3 CNR-ISMN, National Research Council, Institute for the Study of Nanostructured Materials, 00010 Montelibretti, Italy
4 Department of Physics, University of Naples "Federico II", 80126 Napoli, Italy
5 Laboratory of Petrochemical Processes, Institute of Combustion Problems, 050012 Almaty, Kazakhstan; aliakenzhik@gmail.com (A.K.); erdos.ongarbaev@kaznu.edu.kz (Y.O.)
6 Faculty of Chemistry and Chemical Technology, Al-Farabi Kazakh National University, 050040 Almaty, Kazakhstan
7 Faculty of Physics, Adam Mickiewicz University, 62-614 Poznan, Poland; mikolaj.pochylski@amu.edu.pl (M.P.), jacek.gapinski@amu.edu.pl (J.G.)
* Correspondence: pietro.calandra@cnr.it

**Feedstocks characterization (chemical composition and thermal behavior)**

*Feedstocks chemical composition*

Physicochemical properties of biomass including ash content, volatile content, elemental composition (C,H,N,S,O), energy content and chemical composition are key parameters to be known prior to designing any thermochemical biomass modification method as they significantly influence the process conversion performance and the properties of the resulting char. All physicochemical properties of a biomass and their characterization techniques have been reviewed in the paper of Cai et al [Renewable and Sustainable Energy Reviews 76 (2017) 309–322, http://dx.doi.org/10.1016/j.rser.2017.03.072]. Following the recommendations in the review and in accordance with ASTM standards, the feedstocks used as precursors of the char employed in rheological tests were characterized by proximate and ultimate analysis. Additionally, an inductively coupled plasma-mass spectrometry (ICP-MS) analysis was carried out to understand the chemical composition of the inorganic part present in each feedstock.

Table S1 reports the results of proximate analysis performed on all the feedstocks, while Table S2 reports the contents of C, H and N estimated by ultimate analysis and the contents of Na, K, Ca, Mg, and P estimated by inductively coupled plasma-mass spectrometry (ICP-MS) analysis in all feedstocks.

**Table S1.** Feedstocks proximate analysis results.

| | Humidity (wt.%) | Volatiles (wt.%) | Ashes (wt.%) | Fixed carbon (wt.%) | Char@550°C *(wt.%)* |
|---|---|---|---|---|---|
| Lemon peels (LP) | 1.75 | 76.2 | 5.65 | 16.4 | 27.5 |
| Alkali lignin (AL) | 3.80 | 58.3 | 17.6 | 20.3 | 62.0 |
| Citrus Pectin (CP) | 1.87 | 75.8 | 4.53 | 17.8 | 32.1 |
| Shrimp chitosan (SC) | 1.79 | 71.8 | 0.41 | 26.0 | 35.6 |
| Thistle (TH) | 0.46 | 74.7 | 4.84 | 20.0 | 33.3 |

* Evaluated by TG.

**Table S2.** Feedstocks composition.

| | Ultimate analysis | | | | ICP-MS analysis | | | | |
|---|---|---|---|---|---|---|---|---|---|
| | C (wt.%) | H (wt.%) | N (wt.%) | Other elements (wt.%) | Na (wt.%) | K (wt.%) | Ca (wt.%) | Mg (wt.%) | P (wt.%) |
| Lemon peels (LP) | 39.2 | 5.20 | 1.10 | 54.5 | 0.02 | 1.10 | 0.30 | 0.17 | 0.19 |
| Alkali lignin (AL) | 46.6 | 3.70 | - | 49.7 | 4.71 | 0.01 | . | 0.63 | - |
| Citrus Pectin (CP) | 40.2 | 4.10 | - | 55.7 | 1.17 | 0.14 | 0.03 | 0.04 | 0.01 |
| Shrimp chitosan (SC) | 38.0 | 6.32 | 5.08 | 50.6 | 0.11 | - | 0.01 | - | 0.01 |
| Thistle (TH) | 47.7 | 6.10 | 2.80 | 43.4 | 0 | 0.52 | 0.16 | 0.44 | 0.91 |

All the feedstocks are characterized by a high volatile content and a fixed carbon content between 15-30 wt.%. in line with the most of lignocellulosic biomass. A great variability is detected in the ash

contents; SC can be considered a material free of ashes while AL is the feedstock with the highest content of ashes and this characteristic is a consequence of its producing process implementing different acidic and basic reagents.

As concerns the elemental composition, all the feedstocks are very rich in carbon and hydrogen and only SC and LP contain a not negligible amount of nitrogen. The inorganic matter present in the feedstocks is made mainly by alkaline and alkaline earth metals compounds, a feature typical of lignocellulosic biomass [1].

It is important to pointed out the difference between the carbon contents reported into the two tables: the fixed carbon estimated by proximate analysis is the amount of solid combustible residue that remains after biomass is heated and the volatile matter is expelled, while the content of carbon estimated by ultimate analysis is the whole carbon present in the analyzed biomass, namely the carbon content of both organic and inorganic matter (volatiles included).

*Feedstocks thermal behaviour*

Figure S1a-b reports the thermogravimetric analysis (TGA) profiles of the feedstocks whereas Figure 1c-d reports the corresponding derivative thermogravimetric analysis (DTG) curves. For clarity's sake, the curves are grouped according to their essential chemical characteristics: polysaccharidic-rich feedstocks (LP, CP and SC) are reported in left panels (a and c) whereas the lignocellulosic based feedstocks (AL, TH) are reported in right panels (b and d).

LP and CP are the two feedstocks that are less thermally stable (their decomposition starts at a temperature below 200°C), while the other feedstocks decompose at temperatures higher than 250°C. Overall, the thermal decomposition leading to the production of a solid residue (char) can be considered concluded at 550°C for all the feedstocks

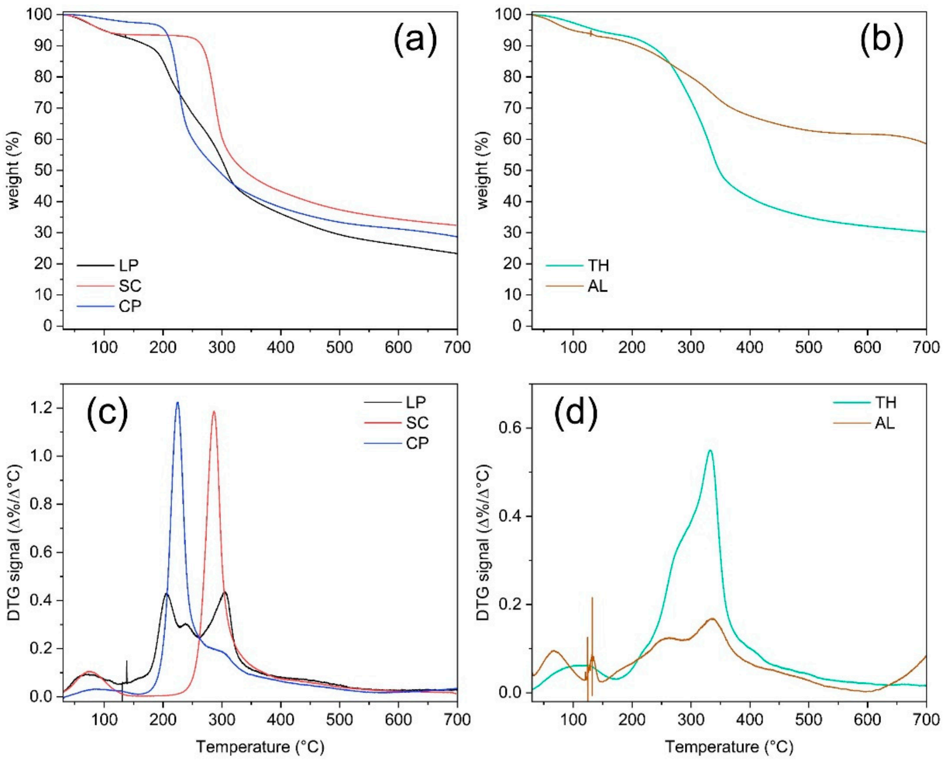


**Figure S1.** TG and DTG curves of the feedstocks ($N_2$, 40 mL/min, HR=5°C/min). Panel a and c TG profiles and DTG curves of polysaccharidic-rich feedstocks (LP, CP and SC). Panels b and d TG profiles and DTG curves of lignocellulosic based feedstocks (AL and TH).

As expected, higher amounts of solid residue (biochar) are given by the feedstocks richer in lignin (AL and TH) and are those more thermally stable. The amounts of solid residue (char) expected on the basis of the TG curve of the feedstocks are reported for each feedstock in Table S1 of Supporting Information. The expected char amount at 550°C has been extrapolated from the TG curves and reported in Table S1 as Char@550°C*(wt.%).

**Char Crystallinity**

The crystallinity of the char samples was probed by XRD analysis, and the resulting patterns are reported in Figure S2.

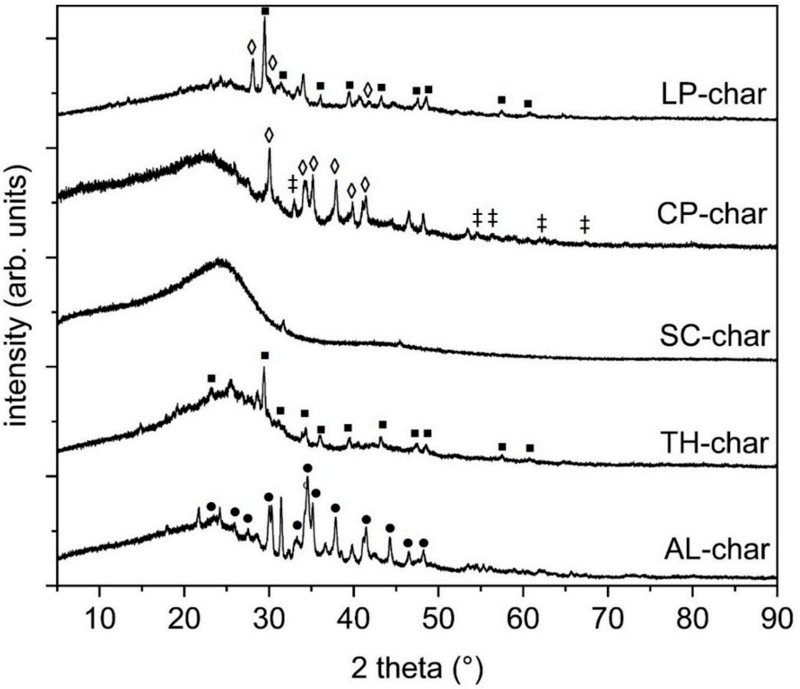


**Figure S2:** XRD patterns of char samples. The symbols are referred to the different phases identified: (◊) Potassium Carbonate (K2CO3, ICDD PDF5 ref. code number: 00-049-1093); (‡) Magnesite (MgCO3, ICDD PDF5 ref. code number: 00-003-0773); (▪) Calcite (CaCO3, ICDD PDF5 ref. code number: 00-005-0586); (●) Natrite (Na2CO3, ICDD PDF5 ref. code number: 00-019-1130).

SC-char and RH-char exhibit a XRD pattern typical of an amorphous carbon. The XRD pattern of SC-char contains a main broad peak around 25 2θ° attributed to SiO2. The XRD patterns of CP-char, TH-char and LP-char contains a broad peak around 25 2θ° ascribable to amorphous carbon and sharper peaks at higher values of 2θ° ascribable to inorganic components. In the case of LP-char and TH-char, calcite (CaCO3) is the main inorganic component identified while in the case of CP-char, natrite (Na2CO3) is the main inorganic component identified.

**Char Surface chemistry**

The surface chemistry of the char samples was investigated by infrared spectroscopy. The FTIR are reported in Figure S3. AL-char FTIR spectrum is characterized by a band peaked around 1500 cm-1 ascribable to the skeletal vibration of C-C and/or C-H bonds, and small intense band peaked around 1580 cm-1 ascribable to the vibration of C=C and C=O bonds. In addition, less intense bands below 1000 cm-1 ascribable to vibrational modes of inorganic components are detected. LP-char, TH-char and CP-char FTIR spectra are quite similar containing broad overlapped bands around 1580 cm-1 ascribable to the skeletal vibration of C=C and/or C=O bonds, an intense peak around 1480 cm-1 ascribable to vibrations of C-C and C-O bonds and low intensity bands below 1000 cm-1 attributable to vibrational modes of inorganic components. The FTIR spectrum of SC-char is characterized by a broad band in the 3000–3700 cm-1 range related to O-H stretching vibrations (possible adsorbed H2O), overlapping bands at 1100–1600 cm-1 due to the skeletal vibration of C-C, C=C and C=O bonds of the carbonaceous network and low intense bands between 700-1000 cm-1 ascribable to the bending of aromatic out of plane C-H bonds. In the SC-char spectrum of note is the presence of a small peak around 2200 cm-1 ascribable to N-containing groups.

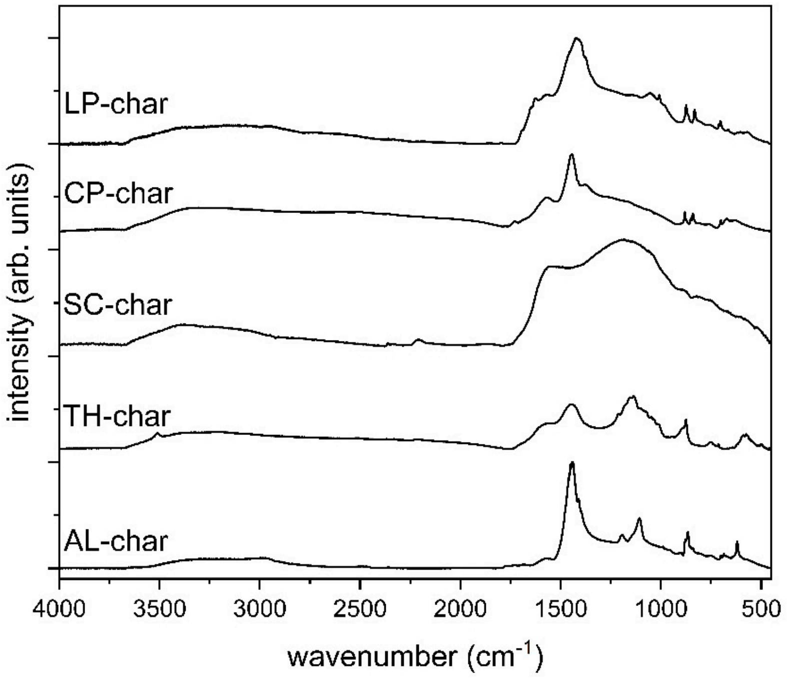


**Figure S3:** FTIR spectra of char samples. The spectra are height-normalized and vertically shifted.

(1) Giudicianni, P.; Gargiulo, V.; Grottola, C. M.; Alfè, M.; Ferreiro, A. I.; Mendes, M. A. A.; Fagnano, M.; Ragucci, R. Inherent Metal Elements in Biomass Pyrolysis: A Review. *Energy & Fuels* **2021**, *35* (7), 5407–5478. https://doi.org/10.1021/acs.energyfuels.0c04046.
